# A Mixture of Phenolic Metabolites of Quercetin Can Decrease Elevated Blood Pressure of Spontaneously Hypertensive Rats Even in Low Doses

**DOI:** 10.3390/nu12010213

**Published:** 2020-01-14

**Authors:** Iveta Najmanová, Jana Pourová, Přemysl Mladěnka

**Affiliations:** 1 Department of Biological and Medical Sciences, Faculty of Pharmacy in Hradec Králové, Charles University, Akademika Heyrovskeho 1203/8, 500 05 Hradec Králové, Czech Republic; najmanoi@faf.cuni.cz; 2 Department of Pharmacology and Toxicology, Faculty of Pharmacy in Hradec Králové, Charles University, Akademika Heyrovskeho 1203/8, 500 05 Hradec Králové, Czech Republic; pourova@faf.cuni.cz

**Keywords:** phenolic, infusion, quercetin, blood pressure, in vivo, rat

## Abstract

Quercetin is proven to decrease arterial blood pressure when given orally. Its bioavailability is, however, low and, therefore, its metabolites could rather be responsible for this effect. In particular, the colonic metabolites of quercetin, 3,4-dihydroxyphenylacetic acid (DHPA), 4-methylcatechol (4MC), and 3-(3-hydroxyphenyl)propionic acid (3HPPA), have been previously shown to decrease the blood pressure in spontaneously hypertensive rats (SHR). Interestingly, the mechanisms of action of these three metabolites are different. The aim of this study is hence to investigate if these metabolites can potentiate each other and thus decrease blood pressure in reduced doses. Three double-combinations of previously mentioned metabolites were administered to SHR as infusions to mimic a real biological situation. All combinations significantly decreased the blood pressure in SHR but there were important differences. The effect of DHPA and 4MC was mild and very short. A combination of DHPA with 3HPPA caused more pronounced effects, which were also rather short-lived. The last combination of 3HPPA and 4MC caused a long-lasting effect. In conclusion, certain combinations of quercetin metabolites have a more pronounced antihypertensive effect than single metabolites.

## 1. Introduction

It is well known that some flavonoids administered orally can decrease arterial blood pressure [1,2]. The bioavailability of parent flavonoids is however low [3] and hence the observed effect could be mediated by their metabolites [4,5]. Although there are some data on conjugates of flavonoids [6], colonic metabolites seem to be more active vasodilators, as was shown recently ex vivo as well as in vivo in spontaneously hypertensive rats (SHR) [7,8]. The most active compounds were 3-(3-hydroxyphenyl)propionic acid (3HPPA), 3,4-dihydroxyphenylacetic acid (DHPA), and 4-methylcatechol (4MC), and interestingly their mechanisms of action were different [7,8]. Data on their pharmacokinetics are limited, but available studies have shown that at least in the cases of 3HPPA and a sulfate conjugate of 4MC, their plasma levels can reach units of µM after administration of a polyphenol reach diet [9,10,11,12]. Previous pharmacodynamic studies were designed to test the effects of single metabolites. In continuation of our research, we decided to test three double-combinations of these most active metabolites in reduced doses to investigate whether they could produce a synergic vasodilatory effect.

## 2. Materials and Methods

DHPA and 4MC were purchased from Sigma-Aldrich (Germany), 3HPPA was from Toronto Research Chemicals (Canada).

Male SHR were obtained from The Czech Academy of Sciences (Czech Republic). The animals were bred in the animal house of the Faculty of Pharmacy and maintained at a constant temperature of 23–25 °C with the 12-h dark/light cycle. Rats were provided a standard diet and tap water ad libitum. The study (reg. No. MSMT-7041/2014-10) was approved by the Ministry of Education, Youth and Sports, and conformed to The Guide for the Care and Use of Laboratory Animals published by the US National Institutes of Health (8th edition, revised 2011, ISBN-13: 978-0-309-15400-0).

Eleven SHR (average weight 381 ± 24 g, blood pressure values under anaesthesia 178 ± 23 mm Hg) were anaesthetised i.p. by pentobarbital 50 mg·kg^−1^. The pressure transducer MLT0380/D was connected with the left common carotid artery, and blood pressure and heart rate were recorded by a Power Lab device with LabChart 7 software (AdInstruments, Sydney, Australia). The combinations of metabolites were administered by an infusion pump (“Genie” Kent syringe pump, Kent Scientific Corporation, Torrington, CT, USA) into the left saphenous vein. Metabolites were dissolved in saline and administered as 5 min infusions (rate 50 μL per minute) in the total doses of 0.25 (i.e., 0.125 + 0.125), 1 (0.5 + 0.5) and 5 (2.5 + 2.5) mg·kg^−1^·min^−1^ in following combinations: DHPA + 4MC (*n* = 3), 3HPPA + DHPA (*n* = 3), and 3HPPA + 4MC (*n* = 5). The next dose was always given after blood pressure was stable for at least 5 min.

## 3. Results and Discussion

**Figure 1:**
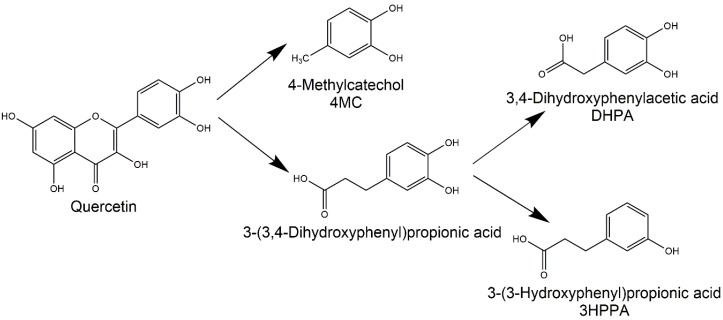
Simplified scheme of the formation of tested metabolites during the degradation of quercetin by microflora in the colon.

**Figure 2:**
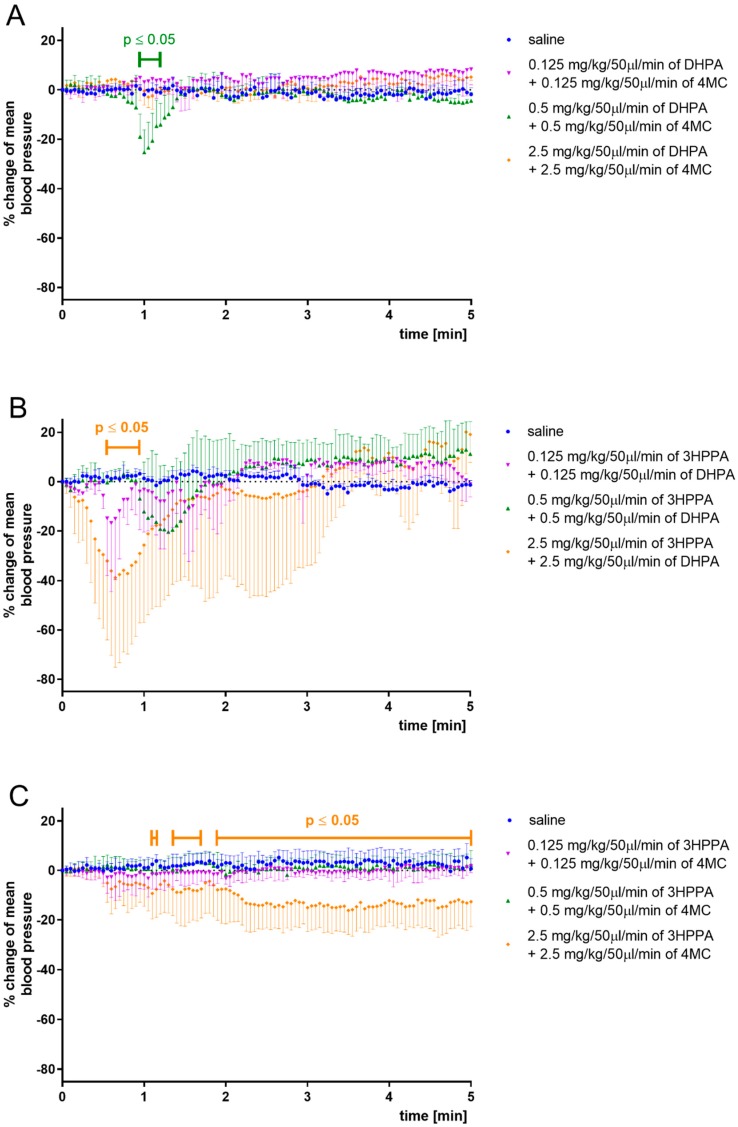
Changes in mean arterial blood pressure after application of 3,4-dihydroxyphenylacetic acid + 4-methylcatechol (DHPA + 4MC) (**A**), 3-(3-hydroxyphenyl)propionic acid + 3,4-dihydroxyphenylacetic acid (3HPPA + DHPA) (**B**), and 3-(3-hydroxyphenyl)propionic acid + 4-methylcatechol (3HPPA + 4MC) (**C**) combinations given as short-lasting infusions. Data are expressed as a mean ± SD.

The DHPA, 4MC, and 3HPPA are three colonic metabolites of quercetin (Figure 1) which have been shown to decrease blood pressure in vivo in SHR. A simultaneous ex vivo mechanistic study has shown that their mechanisms of action are however different [7,8]. Thus, we hypothesized that their combinations could be possibly synergistic. This assumption was found to be partly correct. The combination of DHPA and 4MC (Figure 2A) provoked a significant drop of blood pressure with quick onset (after 1 min) and with a very short duration (15 s). The effective dose (1 mg·kg^−1^·min^−1^) was lower in comparison to their separate administration (the dose of 5 mg·kg^−1^·min^−1^ was needed to produce a hypotensive effect) [8]. Surprisingly, the same combination in the highest dose (5 mg·kg^−1^·min^−1^) did not lead to changes in blood pressure. The reason stays unclear; perhaps this could be due to the desensitization since the infusions were given in a consecutive way in increasing doses to the same animals.

The combination of 3HPPA and DHPA (Figure 2B) at the highest dose (5 mg·kg^−1^·min^−1^) produced a significant decrease in blood pressure. This effect was fast, with a maximum achieved during the first minute since the infusion started. It produced the largest blood decreasing effect from all combinations tested, but again of a short duration (approx. 30 s). Moreover, the values not only quickly returned to the basal levels but slightly increased in some cases. The lower doses (0.25 and 1 mg·kg^−1^·min^−1^) led to similar changes without statistical significance.

Only the last combination of 4MC and 3HPPA (Figure 2C) provoked a gradual and prolonged decrease in elevated blood pressure. The significant and rapid decrease was observed approximately after 1 min of administration, and if we neglect a few fluctuations, the blood pressure remained decreased within the duration of the infusion. This fact deserves attention because in all our previous experiments (including those with administration of individual metabolites), the effects on blood pressure were always only temporal, followed by compensational recovery. Thus, this is the first case when flavonoid colonic metabolites produced a sustained decrease in blood pressure during their administration (Figure 2).

The observed synergic effect could be explained by different mechanisms of action. Although the mechanisms are not fully elucidated at a molecular level, some differences between individual metabolites are apparent. Experiments ex vivo with rat aorta clearly proved that inhibition of eNOS by L-NAME or disruption of endothelium abolish the vasorelaxant potential of 3HPPA, but the effect was preserved in the case of 4MC. The effects of DHPA were only partially endothelium-dependent, and its ability to relax precontracted aortas was presented only at higher concentrations [7,8]. Further experiments are necessary to elucidate these mechanisms in detail, and thus likely to explain why some combinations are more effective than others. It is of note that the pharmacological activity of 4MC is multifarious probably because it can interfere with Ca-trafficking, as was observed in platelets [13]. It is also of interest that the most potent combination tested in this study is based on compounds with contrasting mechanisms of action since 4MC acts NO- and endothelium-independently while the 3HPPA clearly depends on these factors. Moreover, even metabolic conjugation does not necessarily have to to abolish this activity since as it was very recently showed a sulfo-conjugate of 4MC reversed cardiomyocyte hypertrophy induced by phenylephrine, which is a known vasoconstrictor [14]. Anyway, the question of the small phenolic metabolites derived from colonic metabolism and their conjugates is complex, and cannot be fully resolved in this study. Both small phenolic acids of dietary polyphenols and their conjugates can reach quite high plasma concentrations (even 10–25 µM) [9,10,11]. However, studies analyzing the direct contribution of pure polyphenols are lacking. Few available studies with dietary polyphenolic extracts have demonstrated high variability, probably because of the different composition of these mixtures and interindividual variability in human microflora [12]. Interestingly, without supplementation, fasted plasma levels of 3HPPA were 1.5–2.3 µM while that of DHPA only 6–10 nM. An 8-week-lasting intervention with biscuits enriched in olive pomace increased urinary excretion of 3HPPA from about 20 to 34 µM in 24 h; however, the fasting plasma levels were influenced only insignificantly suggesting its short half-life [12]. In fact, our group reported plasma half-live of 3HPPA after i.v. administration to be only 20–40 min [7]. Contrarily, supplementation brought about a huge increase in plasma levels of DHPA to 0.65 ± 0.49 µM [12]. Similar results with DHPA were observed after supplementation with cranberry juice, where plasma c_max_ was 0.48 ± 14 µM [9]. It is of note that an isomer of 3HPPA, 3-(4-hydroxyphenyl)propionic acid, even reached plasma levels of 26 µM after grape seed polyphenols administration in rats [11]. Concerning the 4-MC, the available data reported the plasma c_max_ of its sulfo-conjugate of 3.50 ± 1.19 µM [9]. We have shown that unconjugated, both 4MC and DHPA have short initial plasma half-lives (5–12 min) according to our two-compartmental analysis, which suggests that they are rapidly distributed [8]. Therefore, low plasma concentrations observed only at some time points cannot automatically mean that these substances are biologically inactive since they can be present in the tissues and mediate the observed effects. Future studies are needed to further investigate the precise mechanism(s) of the action as well as to test the vasorelaxant activity of glucuronides and sulfo-conjugates of the above-mentioned metabolites.

## 4. Conclusions

In summary, all metabolite combinations decreased arterial blood pressure. The first combination tested, DHPA and 4MC, only produced a very short-lasting effect. The combination of 3HPPA and DHPA led to a fast decline in blood pressure, but also to a fast return to baseline levels. In contrast, the combination of 3HPPA and 4MC led to a less pronounced but long-term decrease in arterial blood pressure.
